# Convolutional neural networks for the differentiation between benign and malignant renal tumors with a multicenter international computed tomography dataset

**DOI:** 10.1186/s13244-023-01601-8

**Published:** 2024-01-25

**Authors:** Michail E. Klontzas, Georgios Kalarakis, Emmanouil Koltsakis, Thomas Papathomas, Apostolos H. Karantanas, Antonios Tzortzakakis

**Affiliations:** 1 Department of Medical Imaging, University Hospital of Heraklion, Heraklion, Crete, Greece; 2 Computational BioMedicine Laboratory, Institute of Computer Science, Foundation for Research and Technology (FORTH), Heraklion, Crete, Greece; 3 Department of Radiology, School of Medicine, University of Crete, Voutes Campus, Heraklion, Greece; 4 Department of Diagnostic Radiology, Karolinska University Hospital, Stockholm, Sweden; 5 Division of Radiology, Department for Clinical Science, Intervention and Technology (CLINTEC), Karolinska Institutet, Stockholm, Sweden; 6 Institute of Metabolism and Systems Research, University of Birmingham, Birmingham, UK; 7 Department of Clinical Pathology, Vestre Viken Hospital Trust, Drammen, Norway; 8 Medical Radiation Physics and Nuclear Medicine, Section for Nuclear Medicine, Karolinska University Hospital, 14 186, Huddinge, Stockholm, Sweden

**Keywords:** Renal cell carcinoma, Kidney neoplasms, Deep learning, CT scan (Spiral), Artificial intelligence

## Abstract

**Objectives:**

To use convolutional neural networks (CNNs) for the differentiation between benign and malignant renal tumors using contrast-enhanced CT images of a multi-institutional, multi-vendor, and multicenter CT dataset.

**Methods:**

A total of 264 histologically confirmed renal tumors were included, from US and Swedish centers. Images were augmented and divided randomly 70%:30% for algorithm training and testing. Three CNNs (InceptionV3, Inception-ResNetV2, VGG-16) were pretrained with transfer learning and fine-tuned with our dataset to distinguish between malignant and benign tumors. The ensemble consensus decision of the three networks was also recorded. Performance of each network was assessed with receiver operating characteristics (ROC) curves and their area under the curve (AUC-ROC). Saliency maps were created to demonstrate the attention of the highest performing CNN.

**Results:**

Inception-ResNetV2 achieved the highest AUC of 0.918 (95% CI 0.873–0.963), whereas VGG-16 achieved an AUC of 0.813 (95% CI 0.752–0.874). InceptionV3 and ensemble achieved the same performance with an AUC of 0.894 (95% CI 0.844–0.943). Saliency maps indicated that Inception-ResNetV2 decisions are based on the characteristics of the tumor while in most tumors considering the characteristics of the interface between the tumor and the surrounding renal parenchyma.

**Conclusion:**

Deep learning based on a diverse multicenter international dataset can enable accurate differentiation between benign and malignant renal tumors.

**Critical relevance statement:**

Convolutional neural networks trained on a diverse CT dataset can accurately differentiate between benign and malignant renal tumors.

**Key points:**

• Differentiation between benign and malignant tumors based on CT is extremely challenging.

• Inception-ResNetV2 trained on a diverse dataset achieved excellent differentiation between tumor types.

• Deep learning can be used to distinguish between benign and malignant renal tumors.

**Graphical Abstract:**

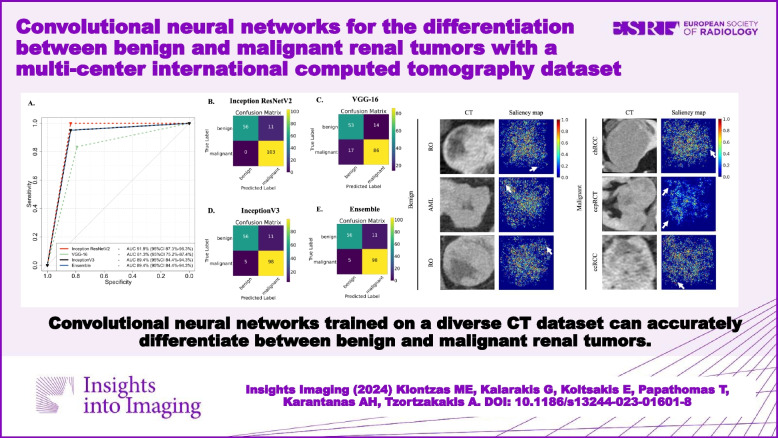

**Supplementary Information:**

The online version contains supplementary material available at 10.1186/s13244-023-01601-8.

## Introduction

Kidney cancer ranks in the 14th place as the most common cancer worldwide, and renal cell carcinoma (RCC) accounts for almost 3% of all cancers, according to the 2022 update of the European Association of Urology [1, 2]. RCC, a heterogenous tumor group, represents 85% of all renal neoplasia, with a hereditary predisposition accounting for 5% of all RCC cases [3]. Differences between genders also exist since RCC contributes 5% of all cancers in males and 3% in females [4]. Despite the absence of screening programs, the increased accidental early detection of renal masses on imaging radiological methods is mainly responsible for the high RCC incidence universally [5]. Mortality rates have lately stabilized in developed countries but continue to rise in developing nations [6]. Epidemiological models predict an increased burden of kidney cancer in the near future [7] associated mainly with risk factors such as chronic/end-stage kidney disease, obesity, smoking, and hypertension [8].

Three primary subcategories of RCC exist, namely clear cell RCC (ccRCC), papillary RCC (pRCC), and chromophobe RCC (chRCC). These subcategories account for approximately 70–80%, 14–17%, and 4–8% of cases, respectively [9]. Regarding metastasis, ccRCC exhibits the highest rate at 8.7%, followed by pRCC at 5.5% and chRCC at 2.9% [10]. Advances in morphologic diagnostic criteria and molecular analyses lead to continuous re-evaluation of renal neoplasia, as reflected in the latest edition (2022) of the World Health Organization (WHO) classification of urogenital neoplasia [11–14]. Based on surgical specimens, renal oncocytoma (RO) accounts for approximately 5% of all renal epithelial neoplasms [15]. As the second benign renal tumor category after angiomyolipoma (AML), RO was falsely considered a malignant tumor for 35 years since its first description in the early 40s [16]. RO and AML lead to unnecessary nephrectomies, as they may exhibit similar radiological imaging characteristics to RCC, contributing to approximately 10% of such cases [17].

The clinical question of reducing the surgical overtreatment of benign renal tumors remains challenging. Conventional radiology cannot definitely differentiate between benign and malignant renal neoplasia due to their similar imaging characteristics [18]. Prominent efforts contributing to a more accurate differentiation of RO from RCC have been reported from modern molecular examination methods, namely ^99m^Tc-sestamibi single-photon emission computed tomography/computed tomography (SPECT/CT) [19] and ^89^Zr-girentuximab positron emission tomography/computed tomography (PET/CT) [20]. A recent pilot study integrating ^99m^Tc-sestamibi SPECT/CT and radiomics via a machine-learning approach reports an accuracy of 95% in detecting renal oncocytic tumors such as RO, HOCT, and low-grade oncocytic tumor (LOT) [21].

Deep learning enables the automation of image recognition without the need for a priori extraction of image features from pre-specified regions of interest [22]. Deep learning has been utilized to study renal neoplasia, mainly focused on the distinction between RCC and RO, disregarding the rest of the tumor subtypes [23]. Studies encompassing data from computed tomography (CT) [23] or magnetic resonance imaging (MRI) [24] report various accuracy rates in the detection of benign renal neoplasia, ranging from 70 to 93.3%. These studies mainly utilize datasets from a single institution with a local patient population which hampers the generalization capacity of the produced algorithms [23]. Xi et al. used a multicenter dataset from a single country that was based on MRI, which is not the first-in-line modality for the evaluation of renal tumors [24]. It is currently widely accepted that single institution datasets cannot capture the complexity of patient, image, and disease patterns, leading to poor generalization capacity of the resulting algorithms [25]. The construction of multi-institutional datasets, including various patient populations that account for different imaging parameters, is complicated due to data privacy issues. Approaches such as federated learning can be used to train algorithms with data from more than one institution, significantly increasing the generalization capacity of the models [26]. Alternatively, open-access data or data from large reference centers that accumulate training images from multiple centers can be utilized [27].

This study aimed to train deep-learning (DL) models that distinguish between benign and malignant renal tumors using contrast-enhanced CT images. Three convoluted neural network (CNN) architectures were trained and tested using a multi-institutional, multi-vendor, and multicenter CT dataset to ensure population diversity and adequate tumor type representation. Developing such a model could reduce the number of falsely detected malignant lesions, thus reducing unnecessary nephrectomies.

## Materials and methods

### Patients

A total of 260 patients were retrospectively included by combining the open-access Kits-19 challenge (https://kits19.grand-challenge.org) training dataset (*n* = 210) collected from a single US-based hospital and patients of the MIDOR dataset (*n* = 50) with examinations collected from 16 hospitals of central Sweden referred for cancer care at Karolinska University Hospital, Huddinge (Stockholm, Sweden). Examinations of the MIDOR dataset were performed in 6 Siemens, 7 Phillips, and 14 General Electric scanners (Supplementary Table 2). This study was conducted in accordance with the Declaration of Helsinki. The MIDOR study was approved by the Karolinska University Hospital (Huddinge) Regional Ethical Review Board and Radiation Safety Committee (2018/1626) [19]. All patients had a histologically confirmed diagnosis either by core biopsy or surgical excision. CT examinations without late arterial images were excluded from the analysis. This multicenter international cohort was used for the training and testing of CNNs. Centers and scanners used for the MIDOR cohort are presented in Supplementary Table 1. The CLAIM checklist ensured that adequate reporting standards were met [28, 29].

### CT imaging and image preparation for deep learning

**Fig. 1:**
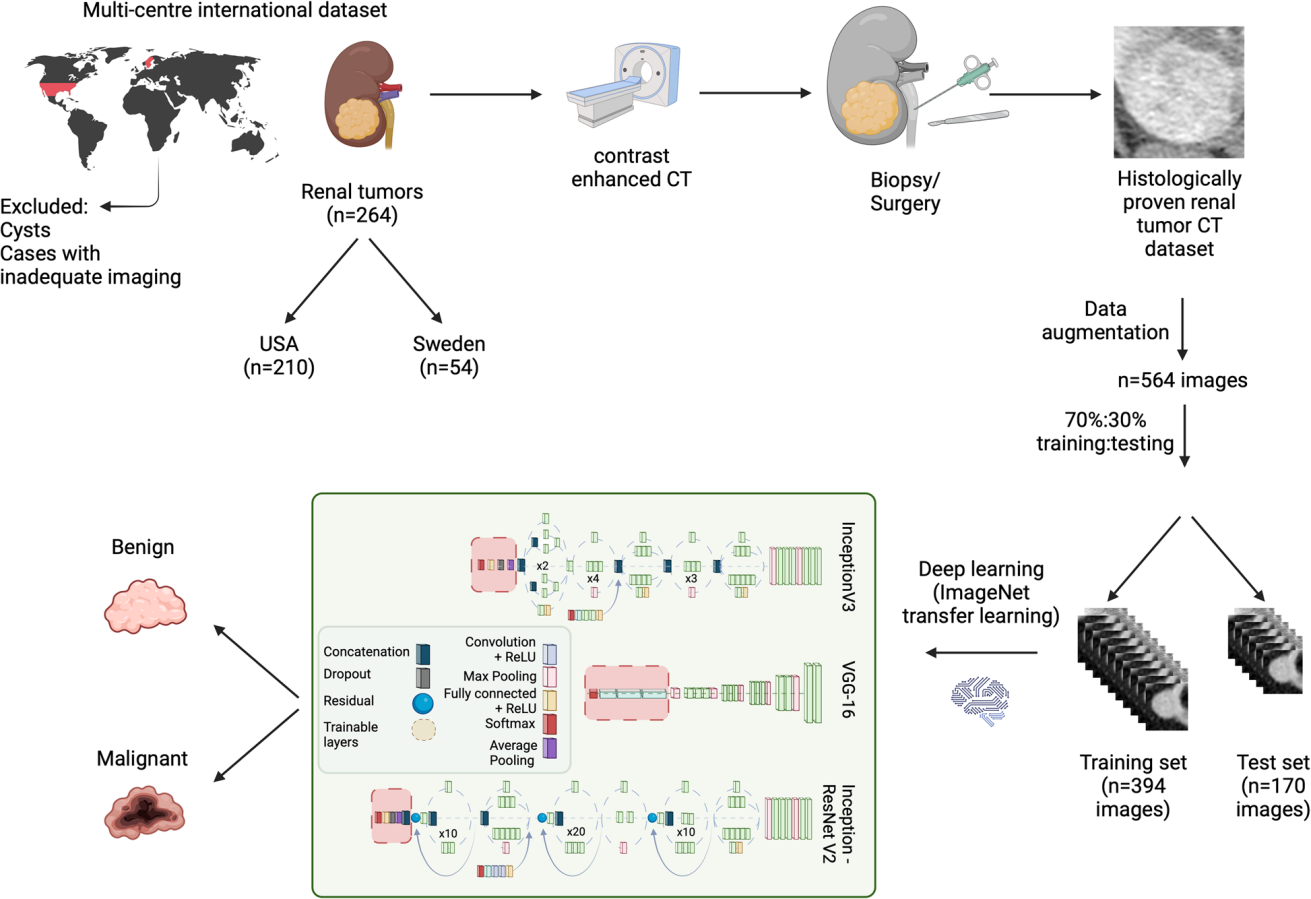
Flow chart demonstrating the data collection, preparation and deep learning process (created with BioRender.com)

Late arterial CT images with a slice thickness of 3 mm were used for DL. In cases a 3-mm slice was not available, this was reconstructed from the existing data. A representative axial section of each tumor was selected and cropped in a rectangular fashion around the tumor, including the interface between the tumor, the surrounding renal parenchyma, and the surrounding fat. Representative images were selected by a consultant radiologist with > 10 years of experience in abdominal imaging. The images selected were usually midsections of the tumors. In case that the tumor was large or contained multiple features (necrotic parts, cystic parts, solid parts, etc.), care was taken to select a slice that included the majority of these features. All images were resized to 150 × 150 pixels for DL model input. Image augmentation was performed using horizontal flipping, 10° clockwise and anticlockwise rotation to reach a total of 564 renal tumor images, which were subsequently randomly split in a 70%:30% ratio for training and testing, respectively, yielding a final dataset of 394 training and 170 testing images (Fig. 1). Data preprocessing and augmentation were performed in Python v3.9.

### Convolutional neural network training and testing

Transfer learning with the ImageNet dataset was used to obtain the initial weights of a VGG-16, an Inception-ResNetV2, and an InceptionV3 CNN with fine-tuning of final layers using our dataset. A consensus ensemble decision of the three CNNs was also recorded as the agreement of at least two out of three CNNs. CNNs were set to be trained with a maximum of 100 epochs using early stopping at *n* = 10 epochs to prevent overfitting. Models were trained with a batch size of 1. Python v3.9 was used for model development using the Keras framework on a MacBook Pro M1 Max 64 GB.

### Model evaluation and statistical analysis

Accuracy, sensitivity (recall), specificity, positive predictive value (PPV precision), negative predictive value (NPV), and f1-scores were calculated to assess the performance of individual CNNs and their consensus ensemble. Receiver operating characteristics (ROC) curves were created to calculate the respective area under the curve (AUC). Two expert readers, one radiology consultant with expertise in urogenital radiology and one senior radiology fellow, evaluated the same images as the models in an attempt to compare the performance of expert readers to the performance of the most accurate of the models presented herein.

Retrospective sample size calculation was performed to estimate the minimum sample required size to detect any AUC-ROC ≥ 0.80 with a 95% CI width ≤ 0.2 given the prevalence of malignant cases in our sample (85%), a power of 80%, and *a* = 0.05. Sample size calculation indicated that a minimum of 103 tumors are needed for such a study.

Integrated gradients saliency maps were produced for tumors of the test group to assess the attention of the CNN with the highest accuracy. Saliency maps indicate the importance of image regions to model performance, offering an important insight on the function of the models while contributing to the interpretability of CNNs. Bootstrapping was used to estimate the 95% confidence intervals AUC-ROC utilizing the pROC R package [30] (R version 4.2.2, https://www.R-project.org/). DeLong’s test [31] was used to compare the AUCs of various CNN models. Statistical significance was defined with a *p*-value less than alpha level = 0.05.

## Results

### Dataset composition

**Fig. 2:**
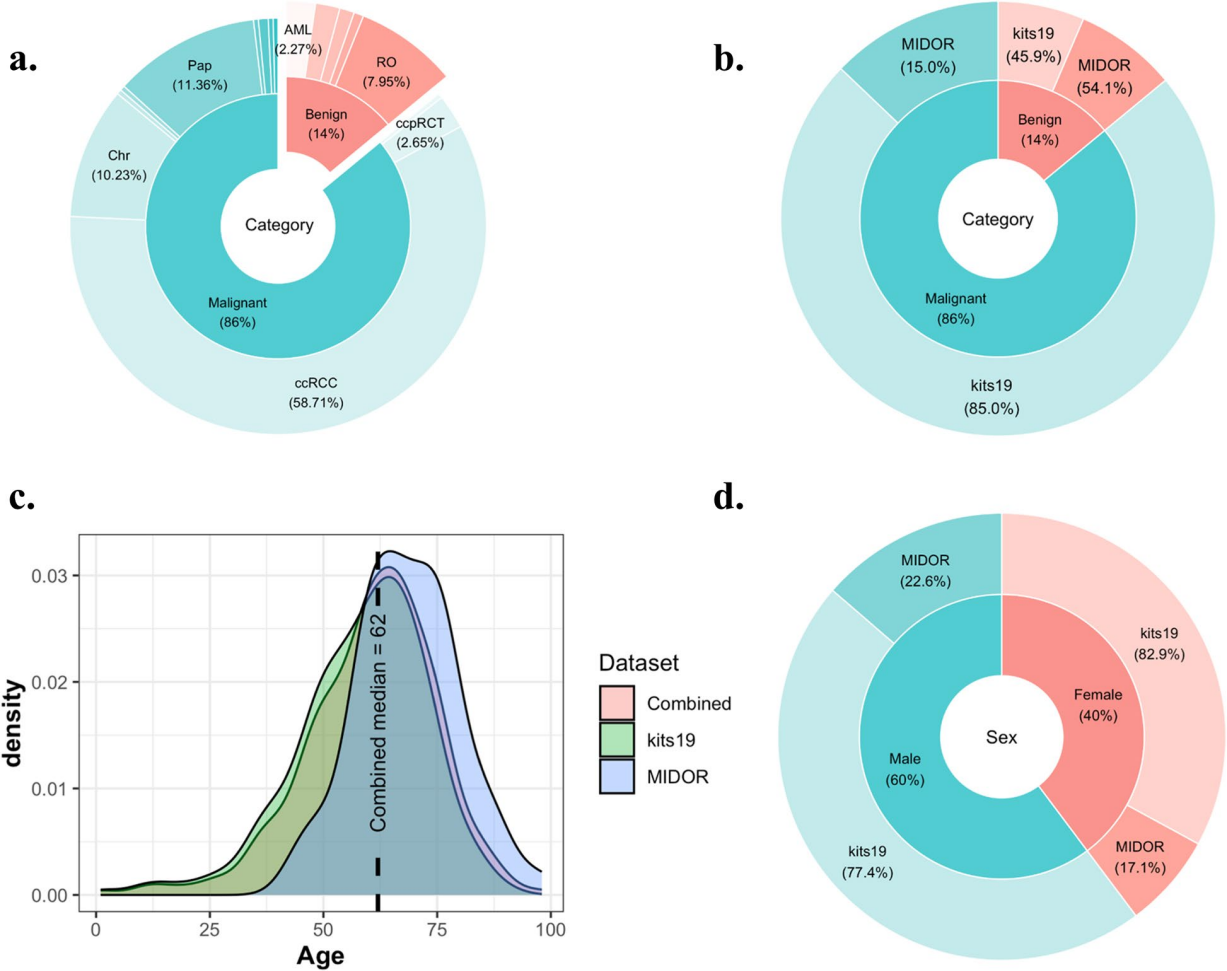
Analysis of the characteristics of our datasets. Pie charts demonstrate the distribution of malignant and benign tumor subtypes (**a**), the distribution of benign and malignant tumors in each one of the sub-datasets (**b**), and the gender of the patients across the MIDOR and kits19 data (**d**). A density plot demonstrates the distribution of patient ages in our data (**c**)

A total of 37 benign (14%) and 227 malignant tumors (86%) were included in the combined dataset. Clear cell RCC comprised the majority of tumors (58.71%), followed by papillary RCC (11.36%) and chromophobes (10.23%) (Fig. 2a). RO comprised only 7.95% of the dataset. The majority of malignant tumors (85%) were included in the kits19 dataset, whereas benign tumors were derived in approximately the same percentage from the kits19 and the MIDOR dataset (45.9% vs 54.1%, respectively) (Fig. 2b). The median age of patients was 62 years, and female and male patients comprised 40% and 60% of the dataset, respectively (Fig. 2c, d). Patient demographics are presented in Supplementary Table 2.

### CNN performance assessment

**Fig. 3:**
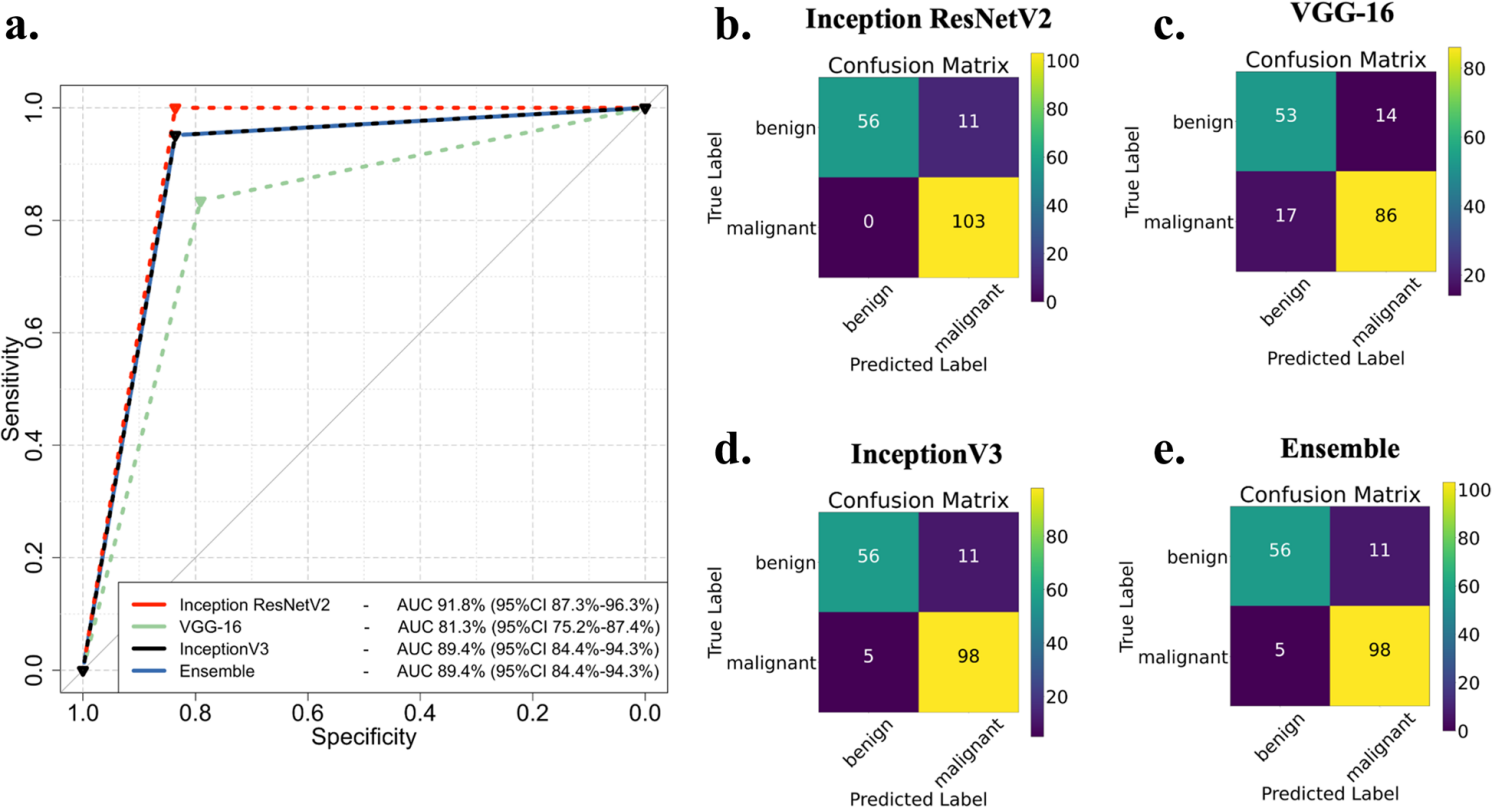
Evaluation of individual convolutional neural networks (CNNs) performance and their ensemble decision. Receiver operating characteristics (ROC) curves of all networks and the ensemble (**a**) and confusion matrices demonstrate the correctly and falsely classified cases for each CNN (**b**–**e**)

**Fig. 4:**
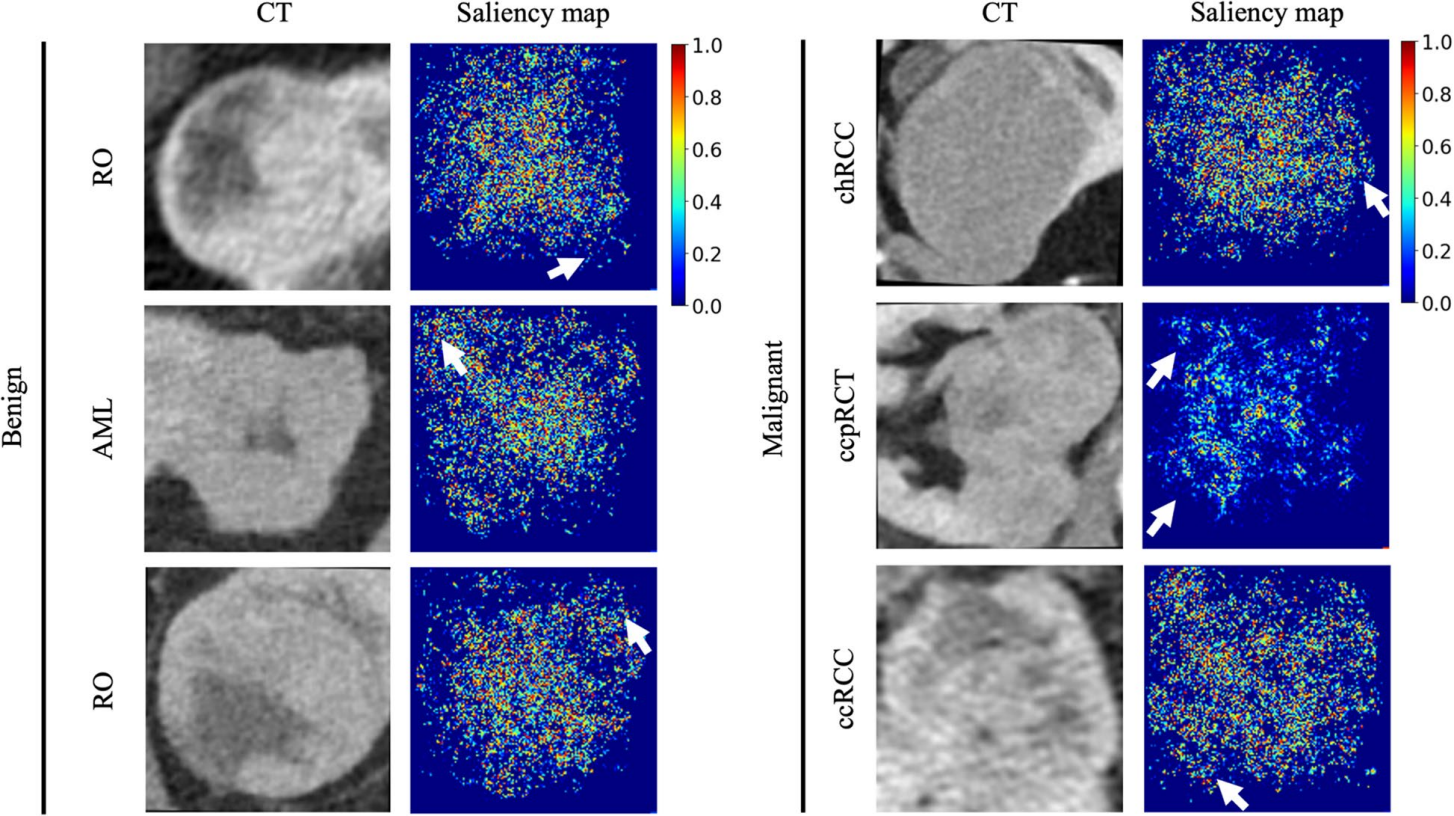
Saliency maps demonstrate the attention of Inception-ResNetV2 for analyzing various malignant and benign tumors. White arrows indicate that the algorithm “looks” either at the surrounding healthy renal parenchyma or peritumoral fat, *RO* Renal oncocytoma, *AML* Angiomyolipoma, *ccRCC* Clear cell RCC, *ccpRCC* Clear cell papillary RCC, *chRCC* Chromophobe RCC

Inception-ResNetV2 achieved the highest AUC of 0.918 (95% CI 0.873–0.963), whereas VGG-16 achieved an AUC of 0.813 (95% CI 0.752–0.874). InceptionV3 and ensemble achieved the same performance with an AUC of 0.894 (95% CI 0.844–0.943) (Fig. 3 and Table 1). Saliency maps of the most important tumor categories indicated that Inception-ResNetV2 decisions were based on the characteristics of the tumor while in most tumors considering the characteristics of the interface between the tumor and the surrounding renal parenchyma. Importantly, saliency maps did not indicate involvement of the peritumoral abdominal fat (Fig. 4). Comparison of saliency maps derived from all three models in benign and malignant tumors indicated that Inception-ResNetV2 selectively focused more selectively on the tumor and peritumoral area compared to InceptionV3 which focused on a more wide area around the tumor. VGG-16 focused weakly in a more wide area of the image without clear concentration at a specific site (Supplementary Fig. 1). It is important to note that Inception-ResNetV2 did not falsely characterize any malignant lesions as benign. Analysis of failed predictions where benign cases were falsely identified as malignant indicated that features such as extracapsular extension, perilesional fat with or without fat stranding, or ill-defined borders of the lesion were areas where Inception-ResNetV2 focused to produce the false-positive decision (Supplementary Fig. 2).

**Table 1:** CNN performance metrics

	**AUC**	**Accuracy**	**Sensitivity (recall)**	**Specificity**	**PPV (precision)**	**NPV**	**f1-score**
**Inception-ResNet-V2**	**91.8% (87.3–96.3%)**	95.18%	90.35%	100%	100%	83.58%	96.6%
**VGG-16**	**81.3% (75.2–87.4%)**	80.86%	86%	75.71%	83.5%	79.1%	84.7%
**InceptionV3**	**89.4% (84.4–94.3%)**	90.86%	89.91%	91.8%	95.15%	83.58%	92.46%
**Model ensemble**	**89.4% (84.4–94.3%)**	90.86%	89.91%	91.8%	95.15%	83.58%	92.46%

*AUC* Area under the curve, *PPV* Positive predictive value, *NPV* Negative predictive value

**Fig. 5:**
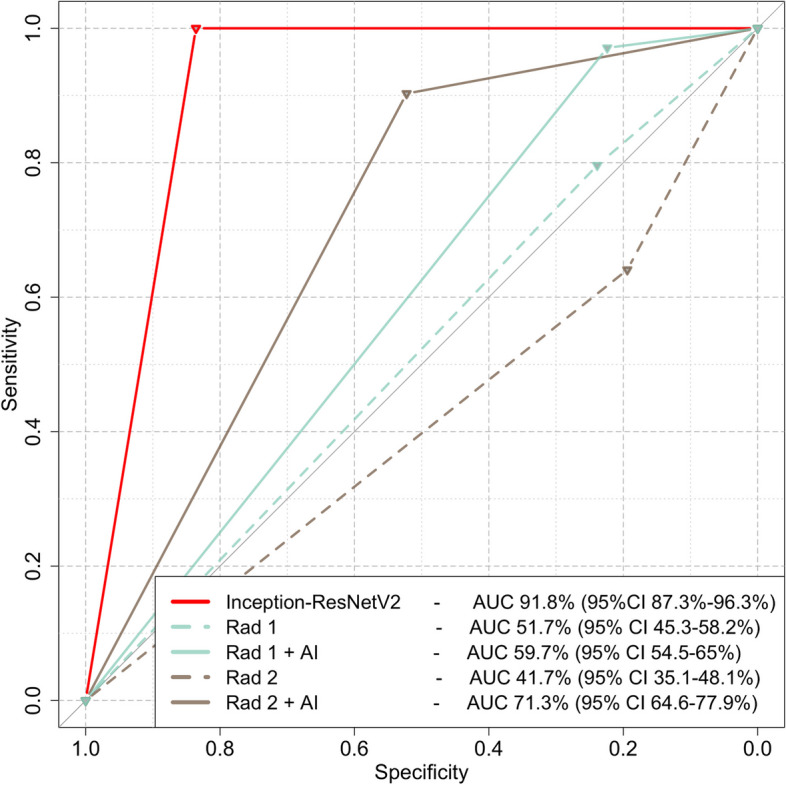
Receiver operating characteristics (ROC) curves of Inception-ResNetV2 compared to the performance of two human readers before (Rad 1 & Rad 2) with and without the help of AI (Rad 1 + AI & Rad 2 + AI). Dashed and continuous lines represent performance without and with AI, respectively. *AUC* Area under the curve, *CI* Confidence interval

The results of the best-performing CNN were compared to the performance of two radiologists, a radiology consultant with experience in abdominal imaging and a senior radiology resident specializing into abdominal imaging. Both radiologists achieved a poor performance with AUCs of 0.517 (95% CI 0.453–0.582) and 41.7% (95% CI 0.351–0.481) for the consultant and senior resident, respectively. The performance of both radiologists was significantly lower than the performance of Inception-ResnetV2 (*p* < 0.001) (Fig. 5). When the radiologists were asked to make the same diagnosis but at this time with access to the prediction of the CNN, both achieved higher performance than without the use of AI (*p* < 0.01) (Fig. 5). To assess the clinical utility of Inception-ResNetV2, a decision curve analysis was performed which showed higher net benefit of the use of Inception-ResNetV2 than the treat-all and treat-none cases at a wide spectrum of threshold probabilities (Supplementary Fig. 3).

## Discussion

Herein, DL was used to distinguish between malignant and benign renal tumors. Three CNNs were trained using a multi-institutional international dataset with Inception-ResNetV2 reaching an excellent performance in the detection of benign renal neoplasia.

Our results are in accordance with a previously published study from Pedersen et al. reporting 90–97.7% accuracy in the detection of RO on CT-derived images [23]. Nonetheless, in their study, only RO was considered a tumor subtype in the benign group. As shown by the distribution of tumors in our dataset, RO is the dominant benign subtype. Still, other benign tumors exist in considerable percentages, possibly hampering the diagnosis. The benign dataset has included the spectrum of available benign tumors. This is also important given that, according to the literature, the percentage of RO and other benign tumors is significantly lower than their malignant counterparts. The ratio benign:malignant tumors in our cohort is similar to the ratio described in other studies and accurately represents the real-world scenario [32]. This group imbalance could cause statistical problems if not accounted during the data preparation and training phase of the DL [22]. In our work, we have mitigated this risk by augmenting the dataset to equalize the number of images between the two groups, a widely used regularization method [33]. Alzubi et al. also used an augmentation process in a dataset of 60 patients with renal malignancy out of 120 patients examined with contrast-enhanced or without contrast-enhanced CT examination from a single-center study reporting an accuracy of 92% in the differentiation of healthy versus tumoral renal parenchyma [34]. In another single-center study by Garner et al. with a dataset of 132 renal lesions examined under the same CT apparat, an accuracy of 87% was reported in a histopathologically verified material [35]. Our study collected histopathologically verified kidney tumors examined in different centra under various CT apparats.

The accurate characterization of renal oncocytic neoplasia is problematic not only in radiological [36] but also on histopathological grounds, especially when obtained from specimens from core biopsies [37]. Patel and colleagues showcased that 25% of RO cases were incorrectly diagnosed, leading to 12.5% and 6.3% of tumors being reclassified as chRCC or HOCT, respectively, after excision [37]. In that aspect, the excellent performance of Inception-ResNetV2 could assist other examination methods that detect renal oncocytic neoplasia, such as ^99m^Tc-sestamibi SPECT/CT [38], to verify or improve their performance.

DL has been previously used to assist in various diagnostic dilemmas related to renal neoplasia. Han et al. achieved an accuracy of 85% in distinguishing between types of malignant tumors, including ccRCC, pRCC, and chRCC [39]. Differentiation between pRCC and chRCC has also been attempted by Zuo et al. [40] achieving an accuracy of ~ 96%. Zheng et al. achieved 60.4% accuracy in differentiating between ccRCC, chRCC, pRCC, and AML [41]. Most studies dealing with benign tumors attempted the distinction between RCC and RO [23, 24, 42, 43]. This distinction disregards the presence of important tumors such as AML and chRCC that can complicate the diagnosis. Oberai et al. focused on the distinction between benign and malignant lipid-poor renal tumors, including lipid-poor angiomyolipoma [44], based on CT with an accuracy of 78%, alas, from a single institution. In our case, Inception-ResNetV2 achieved an accuracy > 90%, an excellent performance, and at the upper limit of performances reporting in literature while examining all available tumor types from a diverse patient population.

The renal parenchyma surrounding the tumor is of utmost importance during tumor resection in the sense that healthy resection margins are always desired. The advent of partial nephrectomy, currently considered the gold standard for early-stage (T1) tumors, has increased the importance of allowing a safe zone around the tumors [45]. Salience maps produced based on our best-performing CNN demonstrated that network attention was focused mainly on the tumor. Some cases also extended marginally to “visually” healthy renal parenchyma. This signifies the current practice of establishing a minimum (at least 4 mm) margins of resection for T1b tumors [46]. In our results, the absence of network attention at the peritumoral abdominal fat potentially indicates that the involvement of the fat is less important than the involvement of the surrounding renal parenchyma in differentiating malignant from benign lesions. The attention of the network at the tumor-renal parenchyma interface may indicate imaging features invisible to the human eye that could be further studied with advanced image analysis methods such as radiomics. Interestingly, assessment of false-positive model predictions (benign cases falsely predicted as malignant) indicated that the model was confused by features which can deceive even experience radiologists, such as ill-defined borders with the normal parenchyma, extracapsular extension, or perilesional fat stranding.

Comparison of our best-performing model to human readers indicated superior performance of the CNN. This is expected given the known inability of human readers to accurately distinguish between benign and malignant renal tumors based on CT. Studies have reported similar results to ours, with specificity of differentiation between benign and malignant tumors around 50% on multiphasic CT [47]. Human readers can use CT to detect the presence of a renal tumor with very high accuracy, approximating 100%. However, distinguishing between benign and malignant lesions is difficult even in cases where MRI is also employed, necessitating biopsy for most of the cases [48]. Importantly, when our readers were asked to make the same diagnosis but with the assistance of AI, their performance was significantly increased, indicating that our CNN can have an important value in everyday clinical practice. Nonetheless, even with the assistance of AI, the performance of humans did not increase higher than 72% which is still not an acceptable diagnostic accuracy for a tumor.

Our study has certain strengths and limitations. Strengths include the diversity of the dataset, the large dataset size relative to other published studies, the pathological confirmation of all cases, and the use of CT images, the most commonly used modality for evaluating such patients. Limitations of our method include its retrospective nature and the lack of tumors that did not undergo resection or biopsy based on their imaging appearance such as renal cysts. The latter could potentially alter the prevalence of benign tumors in our dataset. Nonetheless, the inclusion of pathologically unconfirmed cases would also hamper the trust in our results. Another limitation of our study could be the use only single-phase images. However, despite the use of a single phase, our algorithms exhibited excellent performance. This is in line with previous publications demonstrating that disregarding contrast phase information does not affect the performance of deep learning algorithms in the evaluation of renal tumors [23]. The fact that the developed models were not evaluated on their ability in distinguishing different sub-types of benign or malignant masses is another potential limitation. Nonetheless, this could not be possible given the very small number of certain tumor subtypes. Additionally, the lack of external validation presents one more limitation of this work. The selection of representative images for the model could be a limitation since it could be affected by the experience of the reader. However, identifying the main features of the lesion (e.g., necrosis, cystic components, extracapsular extension) that should also be included in the representative image should be routinely done in any report. It is, therefore, important that readers that are not experienced to report such exams should not be involved in the selection of images. Finally, misclassified cases still exist despite our method’s high performance, which could be potentially reduced by obtaining an even larger dataset.

In conclusion, we demonstrated that CNNs have a high performance in differentiating between benign and malignant renal tumors. The multicenter international dataset used herein ensures the diversity of our training data and represents an important step toward the reproducibility of our algorithms.

### Supplementary Information

**Additional file 1: Supplementary Fig. 1.** Representative saliency maps comparing the attention of Inception-ResNetV2, InceptionV3 and VGG-16. **Supplementary Fig. 2.** Indicative failed predictions of the Inception-ResNetV2 model. All failed cases represent benign tumors falsely labelled as malignant. No malignant tumors of our test set were falsely labelled as benign. Images represent CT appearances of tumors and the respective saliency maps. In the first case (A, B) the algorithm has focused on the extension of the tumor to the surrounding fat (arrows) and has mistaken the fat between the exophytic lesion and the normal parenchuma as part of the tumor (arrowheads). In the second case (C, D) the algorithm has focused on the collecting system and the normal parenchyma (arrows). In the final case (E, F) the arrow has focused on either clear perirenal fat (arrowheads) or at places where lines of fat stranding can be noted (arrows). **Supplementary Fig. 3.** Decision curve analysis demonstrating the clinical value of the best performing model. The standardized net benefit of Inception-ResNetV2 (blue line) is compared to the treat-all (red line) and treat-none (green line) scenarios over the range of threshold probabilities. **Supplementary Table 1.** Centers and equipment used in the MIDOR dataset. **Supplementary Table 2.** Demographics from 50 patients with 54 renal tumors included in the MIDOR study*.

## Data Availability

Kits19 data are available on a CC BY-NC-SA license from https://github.com/neheller/kits19. The rest of the data are available from the corresponding author upon reasonable request.

## Abbreviations

AML: Angiomyolipoma
AUC: Area under the curve
ccRCC: Clear cell RCC
chRCC: Chromophobe RCC
CNN: Convolutional neural network
HOCT: Hybrid oncocytic tumor
LOT: Low-grade oncocytic tumor
pRCC: Papillary RCC
RCC: Renal cell carcinoma
RO: Renal oncocytoma
ROC: Receiver operating characteristics
WHO: World Health Organization
