# Supplementary material for: Convolutional neural networks for the differentiation between benign and malignant renal tumors with a multicenter international computed tomography dataset
Source: Insights Imaging. 2024 Jan 25;15:26. doi: 10.1186/s13244-023-01601-8 (PMC10811309; doi:10.1186/s13244-023-01601-8)
Supplement: Supplementary file 1 — Additional file 1: Supplementary Fig. 1. Representative saliency maps comparing the attention of Inception-ResNetV2, InceptionV3 and VGG-16. Supplementary Fig. 2. Indicative failed predictions of the Inception-ResNetV2 model. All failed cases represent benign tumors falsely labelled as malignant. No malignant tumors of our test set were falsely labelled as benign. Images represent CT appearances of tumors and the respective saliency maps. In the first case (A, B) the algorithm has focused on the extension of the tumor to the surrounding fat (arrows) and has mistaken the fat between the exophytic lesion and the normal parenchuma as part of the tumor (arrowheads). In the second case (C, D) the algorithm has focused on the collecting system and the normal parenchyma (arrows). In the final case (E, F) the arrow has focused on either clear perirenal fat (arrowheads) or at places where lines of fat stranding can be noted (arrows). Supplementary Fig. 3. Decision curve analysis demonstrating the clinical value of the best performing model. The standardized net benefit of Inception-ResNetV2 (blue line) is compared to the treat-all (red line) and treat-none (green line) scenarios over the range of threshold probabilities. Supplementary Table 1. Centers and equipment used in the MIDOR dataset. Supplementary Table 2. Demographics from 50 patients with 54 renal tumors included in the MIDOR study*. [file 13244_2023_1601_MOESM1_ESM.docx]

**Convolutional neural networks for the differentiation between benign and malignant renal tumors with a multi-center international computed tomography dataset**

**ELECTRONIC SUPPLEMENTARY MATERIAL**

**Supplementary Table 1** – Centers and equipment used in the MIDOR dataset

|  | **Karolinska University Hospital** | | **Public Hospitals in Stockholm`s County (2 centers)** | **Private Hospitals in Stockholm`s County (10 centers)** |
| --- | --- | --- | --- | --- |
|  | **Solna** | **Huddinge** |  |  |
| **Siemens Revolution** | 1 |  |  |  |
| **Siemens Somatom** |  | 10 | 1 | 3 |
| **Siemens Sensation** | 2 |  |  |  |
| **Philips Brilliance** |  |  | 2 | 20 |
| **GE Lightspeed** |  |  | 1 | 4 |
| **GE Optima** |  |  |  | 5 |
| **GE Revolution** |  | 1 |  |  |
| **GE Discovery** |  |  | 4 |  |

**Supplementary Table 2** - Demographics from 50 patients with 54 renal tumors included in the MIDOR study*.

| **number of renal tumours** |  | 54 |
| --- | --- | --- |
| **age (median [IQR])** |  | 67.00 [59.00, 75.00] |
| **sex** | female | 15 |
|  | male | 35 |
| **size on CT in mm (median [IQR])** |  | 25.00 [18.00, 40.00] |
| **size categories, mm** | < 20mm | 16 |
|  | > 20mm | 38 |

*Detailed demographics for the Kits-19 dataset can be found in <https://doi.org/10.1016/j.media.2020.101821>

Heller N, Isensee F, Maier-Hein KH, Hou X, Xie C, Li F, Nan Y, Mu G, Lin Z, Han M, Yao G, Gao Y, Zhang Y, Wang Y, Hou F, Yang J, Xiong G, Tian J, Zhong C, Ma J, Rickman J, Dean J, Stai B, Tejpaul R, Oestreich M, Blake P, Kaluzniak H, Raza S, Rosenberg J, Moore K, Walczak E, Rengel Z, Edgerton Z, Vasdev R, Peterson M, McSweeney S, Peterson S, Kalapara A, Sathianathen N, Papanikolopoulos N, Weight C. The state of the art in kidney and kidney tumor segmentation in contrast-enhanced CT imaging: Results of the KiTS19 challenge. Med Image Anal. 2021 Jan;67:101821. doi: 10.1016/j.media.2020.101821. Epub 2020 Oct 2. PMID: 33049579; PMCID: PMC7734203.


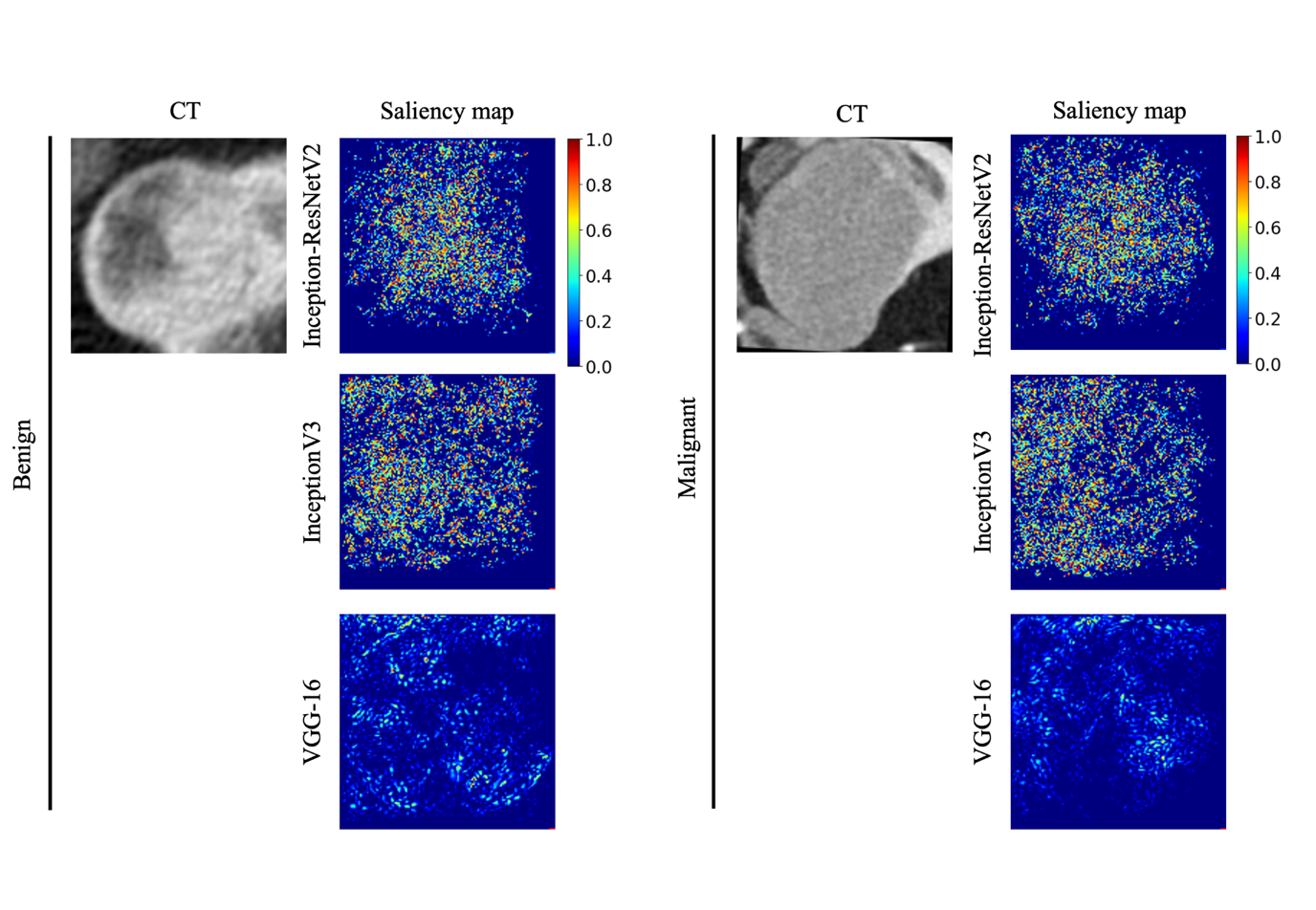


**Supplementary Figure 1**. Representative saliency maps comparing the attention of Inception-ResNetV2, InceptionV3 and VGG-16.


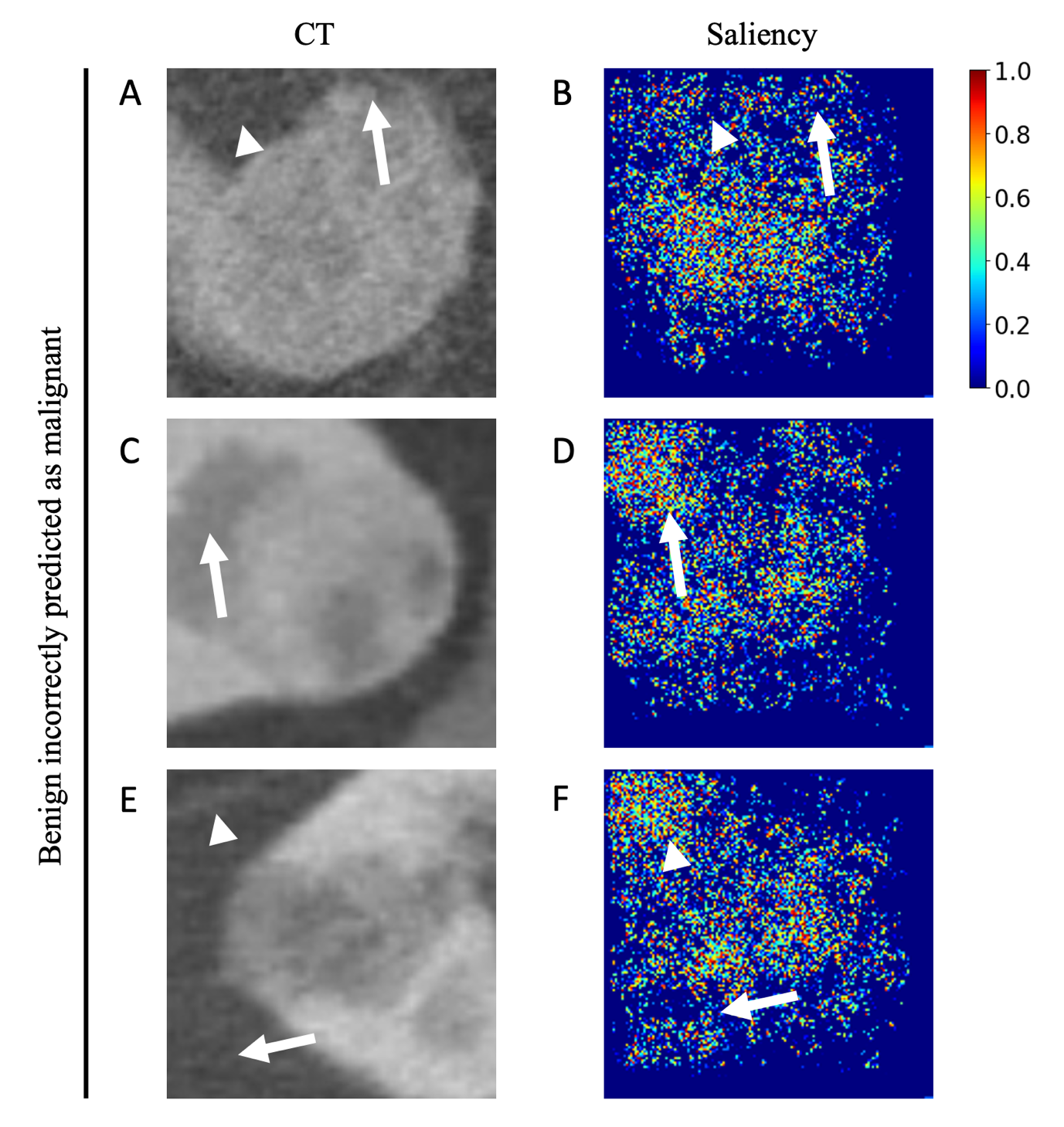


**Supplementary Figure 2**. Indicative failed predictions of the Inception-ResNetV2 model. All failed cases represent benign tumours falsely labelled as malignant. No malignant tumours of our test set were falsely labelled as benign. Images represent CT appearances of tumours and the respective saliency maps. In the first case (A, B) the algorithm has focused on the extension of the tumour to the surrounding fat (arrows) and has mistaken the fat between the exophytic lesion and the normal parenchuma as part of the tumour (arrowheads). In the second case (C, D) the algorithm has focused on the collecting system and the normal parenchyma (arrows). In the final case (E, F) the arrow has focused on either clear perirenal fat (arrowheads) or at places where lines of fat stranding can be noted (arrows).


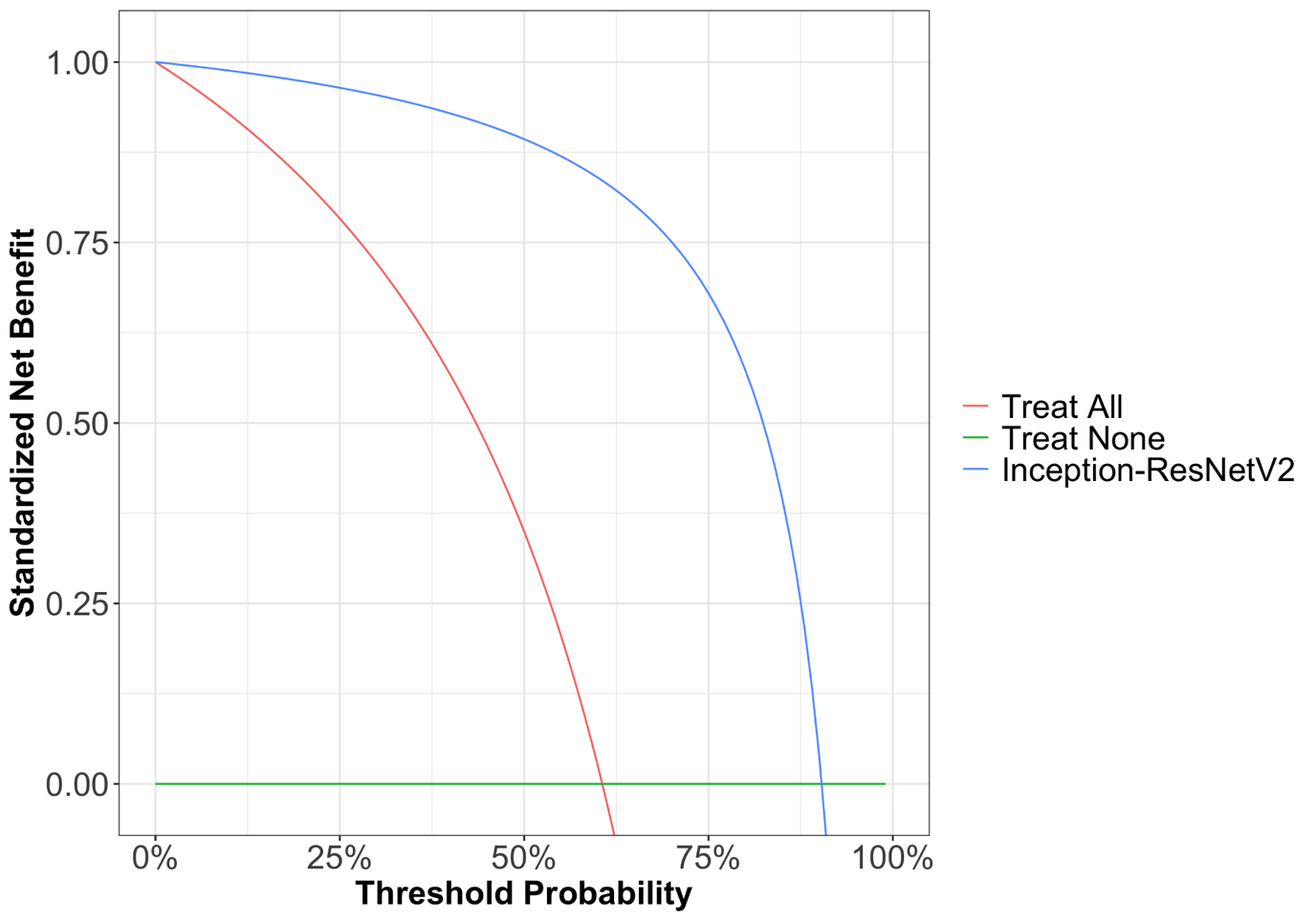


**Supplementary Figure 3**. Decision curve analysis demonstrating the clinical value of the best performing model. The standardized net benefit of Inception-ResNetV2 (blue line) is compared to the treat-all (red line) and treat-none (green line) scenarios over the range of threshold probabilities.

**CLAIM: Checklist for Artificial Intelligence in Medical Imaging**

| Section / Topic | No. | Item |  |
| --- | --- | --- | --- |
| TITLE / ABSTRACT |  |  |  |
|  | **1** | Identification as a study of AI methodology, specifying the category of technology used (e.g., deep learning) | **Yes** |
|  | **2** | Structured summary of study design, methods, results, and conclusions | **Yes** |
| INTRODUCTION |  |  |  |
|  | **3** | Scientific and clinical background, including the intended use and clinical role of the AI approach | **Yes** |
|  | **4** | Study objectives and hypotheses | **Yes** |
| METHODS |  |  |  |
| *Study Design* | **5** | Prospective or retrospective study | **Yes** |
|  | **6** | Study goal, such as model creation, exploratory study, feasibility study, non-inferiority trial | **Yes** |
| *Data* | **7** | Data sources | **Yes** |
|  | **8** | Eligibility criteria: how, where, and when potentially eligible participants or studies were identified (e.g., symptoms, results from previous tests, inclusion in registry, patient-care setting, location, dates) | **Yes** |
|  | **9** | Data pre-processing steps | **Yes** |
|  | **10** | Selection of data subsets, if applicable | **Yes** |
|  | **11** | Definitions of data elements, with references to Common Data Elements | **N/A** |
|  | **12** | De-identification methods | **N/A** |
|  | **13** | How missing data were handled | **N/A** |
| *Ground Truth* | **14** | Definition of ground truth reference standard, in sufficient detail to allow replication | **Yes** |
|  | **15** | Rationale for choosing the reference standard (if alternatives exist) | **No alternatives** |
|  | **16** | Source of ground-truth annotations; qualifications and preparation of annotators | **Yes** |
|  | **17** | Annotation tools | **N/A** |
|  | **18** | Measurement of inter- and intrarater variability; methods to mitigate variability and/or resolve discrepancies | **N/A** |
| *Data Partitions* | **19** | Intended sample size and how it was determined | **Yes** |
|  | **20** | How data were assigned to partitions; specify proportions | **Yes** |
|  | **21** | Level at which partitions are disjoint (e.g., image, study, patient, institution) | **Yes** |
| *Model* | **22** | Detailed description of model, including inputs, outputs, all intermediate layers and connections | **Yes** |
|  | **23** | Software libraries, frameworks, and packages | **Yes** |
|  | **24** | Initialization of model parameters (e.g., randomization, transfer learning) | **Yes** |
| *Training* | **25** | Details of training approach, including data augmentation, hyperparameters, number of models trained | **Yes** |
|  | **26** | Method of selecting the final model | **Yes** |
|  | **27** | Ensembling techniques, if applicable | **Yes** |
| *Evaluation* | **28** | Metrics of model performance | **Yes** |
|  | **29** | Statistical measures of significance and uncertainty (e.g., confidence intervals) | **Yes** |
|  | **30** | Robustness or sensitivity analysis | **Yes** |
|  | **31** | Methods for explainability or interpretability (e.g., saliency maps), and how they were validated | **Yes** |
|  | **32** | Validation or testing on external data | **Np** |
| RESULTS |  |  |  |
| *Data* | **33** | Flow of participants or cases, using a diagram to indicate inclusion and exclusion | **Yes** |
|  | **34** | Demographic and clinical characteristics of cases in each partition | **Yes** |
| *Model performance* | **35** | Performance metrics for optimal model(s) on all data partitions | **Yes** |
|  | **36** | Estimates of diagnostic accuracy and their precision (such as 95% confidence intervals) | **Yes** |
|  | **37** | Failure analysis of incorrectly classified cases |  |
| DISCUSSION |  |  |  |
|  | **38** | Study limitations, including potential bias, statistical uncertainty, and generalizability | **Yes** |
|  | **39** | Implications for practice, including the intended use and/or clinical role | **Yes** |
| OTHER INFORMATION |  |  |  |
|  | **40** | Registration number and name of registry | **N/A** |
|  | **41** | Where the full study protocol can be accessed | **In the text** |
|  | **42** | Sources of funding and other support; role of funders | **Yes** |

Mongan J, Moy L, Kahn CE Jr. Checklist for Artificial Intelligence in Medical Imaging (CLAIM): a guide for authors and reviewers. Radiol Artif Intell 2020; 2(2):e200029. <https://doi.org/10.1148/ryai.2020200029>
